# Clinicodemographic profile of chronic liver disease patients at a tertiary care hospital: a retrospective analysis

**DOI:** 10.1097/MS9.0000000000000248

**Published:** 2023-02-17

**Authors:** Anish K. Shrestha, Anisha Shrestha, Sangam Shah, Aashna Bhandari

**Affiliations:** Institute of Medicine, Tribhuvan University, Maharajgunj, Kathmandu, Nepal

**Keywords:** alcoholic liver disease, chronic liver disease, Nepal, retrospective

## Abstract

**Introduction:**

With the global burden of chronic liver disease (CLD) on the rise, especially due to the rise in obesity and metabolic syndrome, a third-world country like Nepal faces a different problem. With alcohol intake being rooted in Nepalese culture, alcoholic liver disease (ALD) is the most common cause of CLD in our society.

**Methods:**

This is a retrospective observational study conducted in the inpatient ward of the Department of Gastroenterology at the University in Nepal. Ethical approval was taken from the Institutional Review Committee, and a structured questionnaire format was used to record the data retrospectively using admission log books and admission sheets. Demographic data regarding age, sex, and address were collected, while the form of decompensation during presentation was used as a source of clinical data. For statistical analysis, see SPSS 21 (IBM Corp., Released 2012. IBM SPSS Statistics for Windows, Version 21.0; IBM Corp.) was used.

**Results:**

A male-to-female ratio of 2:1 was found, with ALD the most common cause of CLD in admitted patients. Similarly, the majority of patients were admitted due to ascites, which was compounded by spontaneous bacterial peritonitis. 93.60% of patients admitted with CLD had a deranged prothrombin time, while only about a third of patients had elevated aspartate aminotransferase and/or alanine aminotransferase.

**Conclusion:**

The large burden of ALD highlights the importance of awareness programs at the community level, which have been neglected till date.

Highlights

- Alcoholic liver disease contributes to an overwhelming burden of chronic liver disease patients admitted in Nepal.
- Ascites with spontaneous bacterial peritonitis is the most common form of decompensation for which a patient is admitted.
- Awareness programs need to be implemented at the community level to address this issue.

The global burden of chronic liver disease (CLD) is on the rise. Based on data from the Global Burden of Disease study, the age-standardized incidence rate of CLD and cirrhosis was 20.7 per 100 000 in 2015, a 13% increase from 2000^1^. Cirrhosis is among the top 20 causes of disability-adjusted life years^2^. As such, the health and economic burden of CLD with and without cirrhosis is significant globally. Mishra *et al*.,^3^ had shown alcoholic liver disease (ALD) to be the most common cause of CLD in a tertiary care center in Nepal. Our study is aimed at delineating the clinicodemographic profile and assessing the common causes of admission among CLD patients admitted to the inpatient ward of the Department of Gastroenterology at Tribhuvan University Teaching Hospital (TUTH).

## Methods

### Study design and setting

This study was conducted retrospectively at TUTH, located in Maharajgunj, Kathmandu. This center was chosen for study because of its high patient flow. Ethical approval for conducting the study was taken from the Institutional Review Board (IRB) of TUTH, IOM [approval number: 164 (6-11) E^2^ 077/078].

#### Inclusion criteria

Patients with a diagnosis of CLD with or without cirrhosis were admitted to the inpatient ward in the Department of Gastroenterology at TUTH.

#### Exclusion criteria

Patients with acute liver disease.

#### Sampling

Nonprobability sampling.

### Study tools and techniques

A structured proforma was used to record the data retrospectively for the admitted patients.

### Study variables

The variables were categorized under the headings of demographic and clinical factors. Age, sex, and address of the patient were included under demographic factors. Similarly, significant alcohol consumption, as defined by greater than 80 g/day for more than 5 years^4^ and the form of decompensation during their presentation were recorded under clinical factors.

### Statistical analysis

Data were compiled, edited, and checked daily to maintain consistency. The data was collected in Microsoft Excel (Ver. 2013). For statistical analysis, SPSS 21 (IBM Corp. Released 2012. IBM SPSS Statistics for Windows, Version 21.0; IBM Corp.) was used. A descriptive analysis was done to identify the clinicodemographic characteristics of patients.

## Results

**Figure 1:**
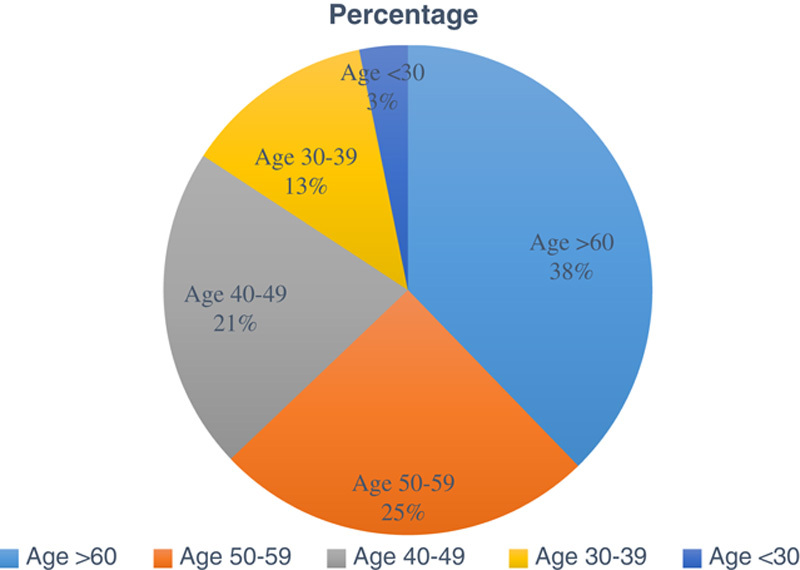
Age-wise distribution of chronic liver disease patients admitted to the inpatient ward.

**Figure 2:**
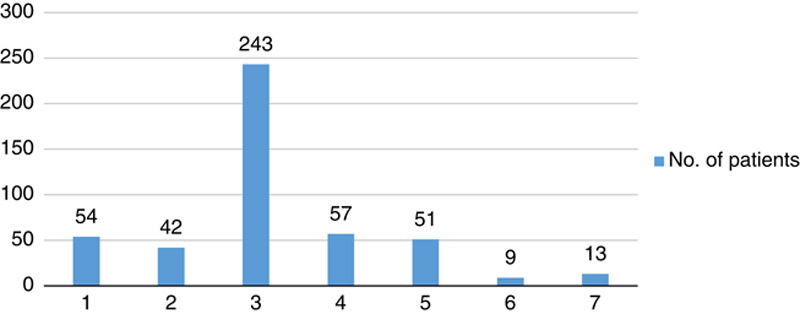
Province-wise distribution of chronic liver disease patients admitted to the inpatient ward.

A total of 469 patients were admitted from January 2018 to December 2019 to the inpatient ward of the Department of Gastroenterology, TUTH, consisting of 303 male (64.60%) and 116 female (35.40%) patients (Table 1 and Figs 1, 2).

**Table 1:** Demographic profile of chronic liver disease patients admitted to the inpatient ward

	*N* (%)
Sex	
Male	303 (64.60)
Female	116 (35.40)
Age	
>60	177 (37.74)
50–59	118 (25.16)
40–49	100 (21.32)
30–39	59 (12.58)
<30	15 (3.20)
Province	
1	54 (11.51)
2	42 (8.96)
3	243 (51.81)
4	57 (12.15)
5	51 (10.88)
6	9 (1.92)
7	13 (2.77)

The majority of patients admitted were from the age group above 60 years (37.74%) and Province 3 (51.81%) of Nepal (Tables 2, 3).

**Table 2:** Clinicolaboratory parameter of patients with CLD

Cause of CLD	
Cause	*N* (%)
Alcohol [alcoholic liver disease (ALD) as defined by intake of >80 g/day for >5 y with CLD features]	386 (82.30)
NAFLD	17 (3.62)
Other causes	66 (14.07)
At admission	
Complication/decompensation	
Ascites	412 (87.84)
Upper gastrointestinal (UGI) bleeding	60 (12.79)
Hepatic encephalopathy (HE)	90 (19.18)
Hepatorenal syndrome/acute kidney injury (HRS/AKI)	51 (10.87)
Spontaneous bacterial peritonitis (SBP)	186 (45.14 of ascites patients)
Laboratory parameters	
Laboratory parameter	
Total bilirubin >21 µmol/l	345 (73.56)
Aspartate aminotransferase (AST) (>2×ULN)	156 (33.26)
Alanine aminotransferase (ALT) [2×ULN (upper limit of normal)]	68 (14.5)
Hemoglobin (<12 g/dl)	363 (77.4)
Platelets (<150 000/mm^3^)	333 (71)
Prothrombin time (deranged, >12 s)	439 (93.60)

CLD, chronic liver disease.

**Table 3:** Laboratory parameters for patients with alcoholic liver disease

Laboratory parameter	*N* (%)
AST/ALT >2	142 (36.79)
AST/ALT ≥1.5	205 (53.11)

ALT, alanine aminotransferase; AST, aspartate aminotransferase; ULN, upper limit normal.

## Discussion

A total of 469 admitted CLD patients were analyzed, with a male-to-female ratio of 2 : 1. Patients aged over 60 years (37.74%) were the ones most commonly admitted for CLD. Since this center lies in Province 3 of Nepal, most patients admitted here were from Province 3 (51.81%), although, being a tertiary care center, a significant number of cases were from other provinces (48.19%) as well.

The absolute number of CLD cases (inclusive of any stage of disease severity) is estimated at 1.5 billion worldwide^1^ with the most common cause being NAFLD (59%), followed by hepatitis B virus (29%), hepatitis C virus (9%), and ALD (2%)^2^,^5^. However, our analysis shows that a major proportion of patients admitted to our center with CLD were due to ALD (82.30%), with other causes contributing to only a minority of cases. The major reason for this is our cultural acceptance of alcohol.

Typical presenting clinical features include jaundice, ascites, hepatic encephalopathy, hepatorenal syndrome, or variceal hemorrhage, which are also the various forms of decompensation in CLD^6^. Among the CLD patients admitted, 65.45% had ascites, 12.79% had upper gastrointestinal bleeding, 19.18% had hepatic encephalopathy, 10.87% had hepatorenal syndrome or acute kidney injury, and 73.56% had jaundice. Similarly, many patients admitted due to ascites as a cause of decompensation had spontaneous bacterial peritonitis (45.14%). This higher percentage of SPB in ascitic patients is due to the rigorous criteria for their admission to the inpatient ward.

In patients with ALD, the aspartate aminotransferase (AST) : alanine aminotransferase (ALT) ratio is greater than 1 in 92% of patients and greater than 2 in 70%^7^. In our study, however, 36.79% of the patients with ALD had an AST/ALT ratio greater than 2, while 53.11% of patients had an AST/ALT ratio greater than 1.5. This could be because of the shorter t1/2 (half-life) of AST (18 h) as compared to ALT (36 h)^8^. Since about 48.19% of patients come to our center from a different province, it is likely that they would have already been treated for a number of days in a different center before their referral here. Therefore, while the AST and ALT levels may be elevated, their ratio may not be greater than or equal to 2:1 over time. Further, although elevated levels of AST and ALT often signify ongoing hepatic inflammation, many patients with CLD may have normal values due to burnout^9^. In such cases, prothrombin time serves as a marker of hepatic function, which still remains elevated.

Anemia is a common finding in CLD patients, with a prevalence ranging from 50 to 87%. The causes of anemia are varied in CLD, ranging from upper gastrointestinal bleeding to anemia of chronic disease, hypersplenism, and malnutrition^10^. About 77.4% of patients in our study had anemia.

This study shows that ALD is the most common cause of CLD in our community reflecting upon the sociocultural acceptance of alcohol in our community. While infectious causes may contribute to a major proportion of cases of acute liver disease, they form only a minority of cases of CLD. Effective vaccination programs and therapeutic options might have contributed to this outcome, though the major contributing factor still seems to be the rampant use of alcohol in our community. The major limitation of the study is the inclusion of only the inpatient population within a single center. Since this a the retrospective study, all the data were not available.

## Conclusion

This study shows the demographic and clinical profile of patients with CLD admitted to the inpatient ward of the Department of Gastroenterology, TUTH. The huge burden of ALD as a contribution to CLD highlights the importance of harm reduction programs that need to be implemented at a community level.

## Ethical approval

Ethical approval was obtained from the research ethics committee of the Institutional Review Committee (IRC) of Institute of Medicine (IOM) [Ref: 164 (6-11) E^2^ 077/078].

## Consent

Written informed consent was obtained from the patient for the publication of this case report and accompanying images. A copy of the written consent is available for review by the Editor-in-Chief of this journal on request.

## Sources of funding

No funding was received for the study.

## Conflicts of interest disclosure

Authors have no conflict of interest to declare.

## Author contribution

A.K.S., A.S., and S.S. wrote the original manuscript, reviewed, and edited the original manuscript. A.K.S., A.S., S.S., and A.B. reviewed and edited the original manuscript.

## Research registration unique identifying number (UIN)

1. Name of the registry: Research Registry.

2. Unique identifying number or registration ID: researchregistry8369.

3. Hyperlink to your specific registration (must be publicly accessible and will be checked): Register Now – Research Registry

## Guarantor

Dr Anish Kumar Shrestha.

## Provenance and peer review

Not commissioned, externally peer-reviewed.

## References

[1] Moon AM Singal AG Tapper EB . Contemporary epidemiology of chronic liver disease and cirrhosis. Clin Gastroenterol Hepatol 2020;18:2650–66. 31401364 10.1016/j.cgh.2019.07.060 PMC7007353

[2] Asrani SK Devarbhavi H Eaton J . Burden of liver diseases in the world. J Hepatol 2019;70:151–71. 30266282 10.1016/j.jhep.2018.09.014

[3] Mishra A Shrestha P Bista N . Pattern of liver diseases. J Nepal Health Res Counc 2009;7:14–18.

[4] Davidson SS . Davidson’s medicine. Davidson’s Princ Pract Med 2010;21:498–9.

[5] Sepanlou SG Safiri S Bisignano C . The global, regional, and national burden of cirrhosis by cause in 195 countries and territories, 1990-2017: a systematic analysis for the Global Burden of Disease Study 2017. Lancet 2020;5:245–66. 10.1016/S2468-1253(19)30349-8 PMC7026710 31981519

[6] Moreau R Jalan R Gines P . Acute-on-chronic liver failure is a distinct syndrome that develops in patients with acute decompensation of cirrhosis. Gastroenterology 2013;144:1426–37. 23474284 10.1053/j.gastro.2013.02.042

[7] Cohen JA Kaplan MM . The SGOT/SGPT ratio – an indicator of alcoholic liver disease. Dig Dis Sci 1979;24:835–38. 520102 10.1007/BF01324898

[8] Botros M Sikaris KA . The De Ritis ratio: the test of time. Clin Biochem Rev 2013;34:117. 24353357 PMC3866949

[9] Lominadze Z Kallwitz ER . Misconception: you can’t have liver disease with normal liver chemistries. Clin Liver Dis 2018;12:96–99. 10.1002/cld.742 PMC6385914 30988921

[10] Scheiner B Semmler G Maurer F . Prevalence of and risk factors for anaemia in patients with advanced chronic liver disease. Liver Int 2020;40:194–204. 31444993 10.1111/liv.14229 PMC6973120

